# Epidemiological Characterization and Genetic Variation of the SARS-CoV-2 Delta Variant in Palestine

**DOI:** 10.3390/pathogens13060521

**Published:** 2024-06-20

**Authors:** Suheir Ereqat, Nabil-Fareed Alikhan, Amer Al-Jawabreh, Michaela Matthews, Ahmed Al-Jawabreh, Leonardo de Oliveira Martins, Alexander J. Trotter, Mai Al-Kaila, Andrew J. Page, Mark J. Pallen, Abedelmajeed Nasereddin

**Affiliations:** 1 Biochemistry and Molecular Biology Department, Faculty of Medicine, Al-Quds University, Abu Dis, Jerusalem P.O. Box 51000, Palestine; sereqat@staff.alquds.edu (S.E.); aal-deen@staff.alquds.edu (A.N.); 2 Quadram Institute Bioscience, Norwich Research Park, Norwich NR4 7UQ, UK; nabil-fareed.alikhan@quadram.ac.uk (N.-F.A.); leonardo.de-oliveira-martins@quadram.ac.uk (L.d.O.M.); alex.trotter@quadram.ac.uk (A.J.T.); andrew.page@quadram.ac.uk (A.J.P.); mark.pallen@quadram.ac.uk (M.J.P.); 3 Department of Medical Laboratory Sciences, Faculty of Allied Health Sciences, Arab American University, Jenin P.O. Box 240, Palestine; 4 Leishmaniases Research Unit, Jericho P5840227, Palestine; 5 Ministry of Health of the State of Palestine, Ramallah P6028100, Palestine; maialkaila@yahoo.it; 6 University of East Anglia, Norwich Research Park, Norwich NR4 7TJ, UK; 7 School of Veterinary Medicine, University of Surrey, Guildford GU2 7AL, UK; 8 Al-Quds Bard College, Al-Quds University, East Jerusalem P.O. Box 20002, Palestine

**Keywords:** SARS-CoV-2, mutation, Palestine, COVID-19, genomic epidemiology

## Abstract

The emergence of new SARS-CoV-2 variants in Palestine highlights the need for continuous genetic surveillance and accurate screening strategies. This case series study aimed to investigate the geographic distribution and genetic variation of the SARS-CoV-2 Delta Variant in Palestine in August 2021. Samples were collected at random in August 2021 (*n* = 571) from eight districts in the West Bank, Palestine. All samples were confirmed as positive for COVID-19 by RT-PCR. The samples passed the quality control test and were successfully sequenced using the ARTIC protocol. The Delta Variant was revealed to have four dominant lineages: B.1.617 (19%), AY.122 (18%), AY.106 (17%), and AY.121 (13%). The study revealed eight significant purely spatial clusters (*p* < 0.005) distributed in the northern and southern parts of Palestine. Phylogenetic analysis of SARS-CoV-2 genomes (*n* = 552) showed no geographically specific clades. The haplotype network revealed three haplogroups without any geographic distribution. Chronologically, the Delta Variant peak in Palestine was shortly preceded by the one in the neighboring Israeli community and shortly followed by the peak in Jordan. In addition, the study revealed an extremely intense transmission network of the Delta Variant circulating between the Palestinian districts as hubs (SHR ≈ 0.5), with Al-Khalil, the district with the highest prevalence of COVID-19, witnessing the highest frequency of transitions. Genetic diversity analysis indicated closely related haplogroups, as haplotype diversity (Hd) is high but has low nucleotide diversity (π). However, nucleotide diversity (π) in Palestine is still higher than the global figures. Neutrality tests were significantly (*p* < 0.05) low, including Tajima’s D, Fu-Li’s F, and Fu-Li’s D, suggesting one or more of the following: population expansion, selective sweep, and natural negative selection. Wright’s F-statistic (Fst) showed genetic differentiation (Fst > 0.25) with low to medium gene flow (Nm). Recombination events were minimal between clusters (R_m_) and between adjacent sites (R_s_). The study confirms the utility of the whole genome sequence as a surveillance system to track the emergence of new SARS-CoV-2 variants for any possible geographical association and the use of genetic variation analysis and haplotype networking to delineate any minimal change or slight deviation in the viral genome from a reference strain.

## 1. Introduction

Coronavirus disease 2019 (COVID-19), caused by severe acute respiratory syndrome coronavirus 2 (SARS-CoV-2), was first reported in Wuhan, PR China in late 2019. In March 2020, the World Health Organization (WHO) declared the COVID-19 outbreak a global pandemic [1]. As of July 2022, there have been over 562 million reported cases and 6.3 million fatalities [2]. Genome sequencing of SARS-CoV-2 positive samples allows for mutations to be identified and variants to be categorized [3,4,5]. Analyses of SARS-CoV-2 genomes have revealed successive “Variants of Concern” (VOCs), characterized by distinctive sets of mutations and renewed epidemic potential, leading to successive waves of the COVID-19 pandemic [6]. Since June 2021, these VOCs have been assigned letters of the Greek alphabet within a WHO naming scheme [7]. The Alpha Variant (PANGO lineage B.1.1.7) was first detected in the UK in September 2020, the Delta Variant (PANGO lineage B.1.617.2) was first detected in India in October 2020, and the Omicron Variant (B.1.1.529) emerged in multiple countries in late 2021 [8]. Emergence of these variants highlights the need for continuous genomic surveillance to identify and track new SARS-CoV-2 variants in real time.

The first cases of COVID-19 were detected in the West Bank, Palestine in March 2020 [9]. By February 2022, over 0.6 million cases and over 5000 deaths had been reported from Palestine [9]. A study conducted in the West Bank, Palestine showed that the country’s first wave in 2020 was caused by close relatives of the original Wuhan strain [10,11]. A subsequent study showed that a second surge of COVID-19 in early 2021 was associated with the Alpha lineage (B.1.1.7) [12]. Here, we have investigated the geographic distribution and genetic diversity of the SARS-CoV-2 Delta Variant whole genomes in Palestine in August 2021 as a prerequisite to elucidating the dynamics of disease transmission and the source of infection in the Palestinian community based on the whole-genome sequencing (WGS) and the observation of mutation dynamics and changes in the viral genome that affect the viral behavior in terms of spread and pathogenicity, in addition to vaccine candidate suitability.

## 2. Materials and Methods

### 2.1. Study Samples and Processing

**Figure 1:**
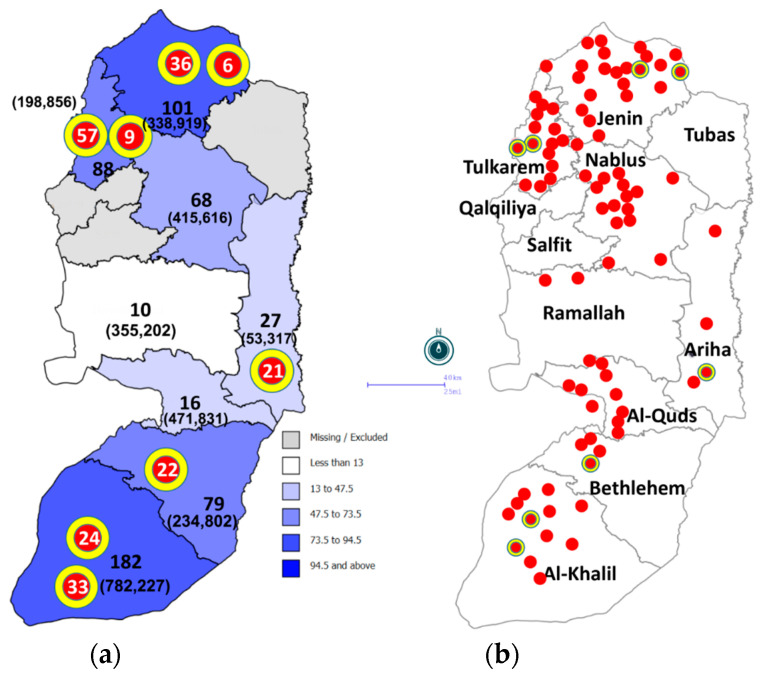
(**a**) Choropleth map showing density of 552 COVID-19 patients per district as indicated by intensity of blue color. The Arabic numerals in black represent the number of COVID-19 samples collected from each district. Numbers in brackets represent the population of the given district in the year 2021. Red circles with yellow rims represent the statistically significant purely spatial clusters of COVID-19 in the study area. (**b**) Spot and cluster map of the distribution of samples by locality represented by red circle, which may contain more than one sample per locality. The eight significant clusters are represented by the red circles with yellow rims.

In this case series study, nasopharyngeal swabs were arbitrarily collected from COVID-19 patients residing in different Palestinian districts in August 2021 during the third wave of the pandemic, without consideration of age, gender, place of residence, or symptoms. The patients’ samples were selected from the Ministry of Health (MOH) central laboratory, which serves as a hub for the satellite laboratories throughout the West Bank (Figure 1) and hold all the samples and data in the country. The patients were non-hospitalized individuals from the Palestinian community seeking COVID-19 testing. Hospitalized individuals, non-Palestinians, and those with incomplete data records were excluded from the study. As the population of the West Bank was 3.15 million, and the expected frequency (prevalence) of COVID-19 was 50%, the sample size at a 95% confidence level was calculated by EpiInfo v. 7.2.4.0 (CDC-free software) to be 384. The assumption of 50% prevalence gives a maximum sample size. Extra samples were included to account any possible test failures (*n* = 571). Viral RNA was extracted from all swabs at the Palestinian Ministry of Health laboratories and confirmed as SARS-CoV-2 cases by real-time (RT) PCR.

cDNA synthesis was performed with LunaScript RT SuperMix (New England Biolabs, Ipswich, MA, USA). DNA library preparation was based on ARTIC v4 amplicon using the IDT ARTIC nCoV-2019 V3 Panel [13] as previously described [14,15]. Libraries were sequenced using the NextSeq 500/550 Mid Output Kit v2.5 (150 Cycles) on the NextSeq 500 platform (Illumina, San Diego, CA, USA) at Quadram Institute Biosciences, Norwich, UK. One positive control containing an extinct lineage (B.1.177) and one negative control were included for each set of 94 samples.

### 2.2. Genome Sequencing and Strain Typing

A total of 571 RNA samples passed the quality control test indicating high viral loads and were successfully sequenced as follows. The raw reads were demultiplexed using bcl2fastq (v2.20) [16]. The reads were used to generate a consensus sequence using the ARTIC bioinformatics pipeline [15]. Briefly, the reads had adapters trimmed with TrimGalore [17] and were aligned to the Wuhan Hu-1 reference genome (Genbank reference accession MN908947.3) using BWA-MEM (v0.7.17) [18]; the ARTIC amplicons were trimmed, and a consensus was built using iVAR (v1.3.0) [19]. PANGO lineages were assigned using Pangolin (v3.1.20 and v4.0.6) [20].

### 2.3. Phylogenetic and Haplotype Analyses

The phylogenetic tree was built from 552 sequences using IQTREE2 (v2.2.0-beta) with a UNREST+FO+I+G4 model, chosen by the IQTREE2’s ModelFinder module [21]. The network illustrating state change between different districts was generated using StrainHub [22]. A parsimony ancestral reconstruction step is performed to create links between the tree and its associated metadata. The transmission network was implemented based on the degree centrality metric, which is the number of edges originating from (outdegree) and ending on (indegree) a given node and the “Source Hub Ratio” (SHR), which represents the ratio of all outward transitions from the node over all transitions from and to the node. A node scoring a SHR close to 1 indicates a source, a SHR close to 0.5 a hub, and a SHR close to 0 a sink for SARS-CoV-2. Temporal data were not used in the construction of this network.

In a second step of analysis, a median-joining haplotype network was constructed using the PopArt 1.7 based on single-nucleotide variation (SNV) across the SARS-CoV-2 S-protein domain spike gene, which contains the Delta Variant mutations that play a pivotal role in the infection process, according to default parameters [10,23,24,25]. Within this context, the term “haplotype” refers to the occurrence of a group of SARS-CoV2 sequences clustering in one unit and differing in at least a single nucleotide (single-nucleotide variation, SNV) to identify patterns of intra-population genetic variation. Along the same line, a “haplogroup’ is a group of closely linked haplotypes clustered together on the network and which tend to be inherited together. The network was constructed based on the geographical origin of the viral isolate. The overall number of DNA sequences (*n* = 552) was filtered by removing all sequences with extensively missing nucleotides, as pointed out by the software.

Based on the assumptions that SARS-CoV-2 has a high mutation rate, probable homoplasy, and a possibility of recombination, the parsimonious phylogenetic tree and median-joining haplotype networking were used side by side to maximize the probability of finding all connections to discern the genetic variation [26].

### 2.4. Genetic Diversity Analyses

The genetic variation analyses were based on the number of haplogroups produced by the haplotype network analysis using the PopArt 1.7. DnaSP ver. 6.12.03 was used to conduct the analyses, which included nucleotide–haplotype diversity, tests of neutrality, genetic differentiation estimators, and the degree of recombination, as described elsewhere [12,27]. Haplotype diversity (Hd or *h*) was calculated by DnaSP ver. 6.12.03 using the following formula:

(1) $$h=n\left(1-\sum {\hat{x}}_{i}^{2}\right)/\left(n-1\right)$$

where *n* is the number of individuals sampled, and *x_i_* is the allele frequency. The hat sign (^) indicates estimation. Nei’s original equation had 2*n* [28].

Assuming that genetic diversity is driven by the processes of recombination, reassortment, and random mutation within the population, the nucleotide diversity, Pi (π) between *i*th and *j*th DNA sequences in the sample, was calculated by DnaSP ver. 6.12.03 using the following formula:

(2) $$\hat{\pi }=\frac{n}{n-1}\sum_{ij}{\hat{x}}_{i}{\hat{x}}_{j}\pi_{ij}$$

where *n* is the number of DNA sequences examined, *x*_*i*_ is the population frequency of the *i*th type of DNA sequence, *x*_*j*_ is the population frequency of the *j*th type of DNA sequence, and $\pi_{ij}$ is the number of nucleotide differences per site between the *i*th and *j*th sequences. The hat sign (^) indicates estimation.

### 2.5. Purely Spatial Cluster Mapping

The choropleth, spot, and cluster mapping of COVID-19 cases from Palestine were constructed with statistical inference using the SaTScan™ v9.7 mapping package for the spatial scan statistics of case clustering and the Epi Info™ 7 statistical package (CDC free-software) for accurate mapping. SaTScan™ v9.7 Freeware was used to detect statistical evidence for purely spatial clustering of cases in the study area. The input files included the number of cases per locality, year of infection, population size of the location in the year of infection, and the exact latitude–longitude coordinates of each location. Data were analyzed based on the discrete Poisson model, using Monte Carlo hypothesis. The level of significance used was at *p*-value ≤ 0.05 [29].

## 3. Results

### 3.1. Study Population

Our study included a total of 571 SARS-CoV-2 samples that were obtained in August 2021 from the Ministry of Health (MOH) Central Laboratory in Ramallah, the West Bank. This center serves as the reference laboratory and receives all COVID-19 positive samples from districts across Palestine excluding the Gaza Strip. The median age of study participants was 27 years, ranging from 1 to 84 years. The sex distribution was 50% females compared to 45% males, with 5% unknown. The samples were from 90 Palestinian localities in eight Palestinian districts, including Bethlehem (13.8%), Al-Khalil (32%), Jenin (18%), Ariha (Jericho) (4.7%), East Jerusalem (Al-Quds) (2.6%), Nablus (12%), Ramallah (2%), and Tulkarem (15%) (Figure 1, Supplementary Table S1). Genome sequences were deposited in the GISAID database (EPI_ISL_10033368-EPI_ISL_10033935).

### 3.2. Identification of Delta Lineages and Sub-Lineages

**Figure 2:**
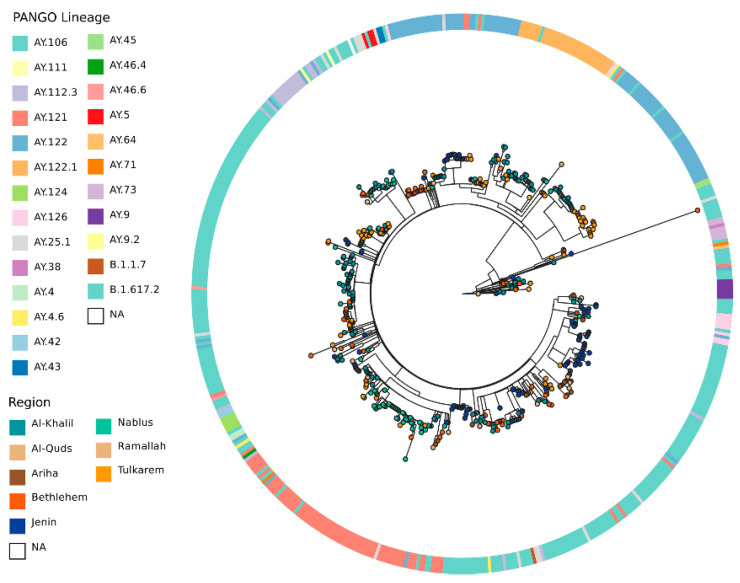
Maximum likelihood phylogenetic tree of 552 SARS-CoV-2 whole-genome sequences obtained from eight geographical regions (districts). Nodes are colored by location of collection, as indicated in the key, and the concentric ring indicates PANGO lineage. Tree was rooted to the midpoint. Full contextual data for samples can be found in Supplementary Table S1.

Our study showed that the most frequent lineage in August 2021 (representing the third wave of the SARS-CoV-2 pandemic) was the Delta Variant, which was detected in 99.9% (*n* = 570) of cases. The frequency of SARS-CoV-2 lineages/sub-lineages is shown in Table 1. Our results showed that 25 AY lineages were circulating in Palestine (the West Bank), with four dominating in the area: B.1.617.2 (31%), AY.122 (16%), AY.106 (19%), and AY.121 (13%). All study districts contained at least seven lineages (Figure 2).

### 3.3. Determination of Distribution and Purely Spatial Clusters

Considering the population size of a given locality and the number of COVID-19 cases within it, the study revealed 16 purely spatial clusters in Palestine (the West Bank). However, only eight of these were statistically significant (*p* < 0.05) (Table 2 and Figure 1). The significant clusters were distributed in the northern and southern parts of Palestine, with the most significant clusters in the village of Beit-Kahil (Al-Khalil district) (RR: 8.2, *p* < 0.001) and Jalboun village (Jenin district) (RR: 6.2, *p* < 0.001). The locality with the largest bulk of COVID-19 samples was the city of Al-Khalil (*n* = 90). However, it did not form a statistically significant cluster.

### 3.4. Phylogenetic and Haplotype Analyses

We constructed a phylogenetic relationship between the Delta Variant genomes (Figure 2). The phylogenetic tree included 552 genome sequences; these 552 out of 571 were those with less than 50% sequence ambiguity. Delta Variant genomes represent a diverse range of lineages consistent with the PANGO lineages defined (*n* = 25). No geographically specific clades were defined.

**Figure 3:**
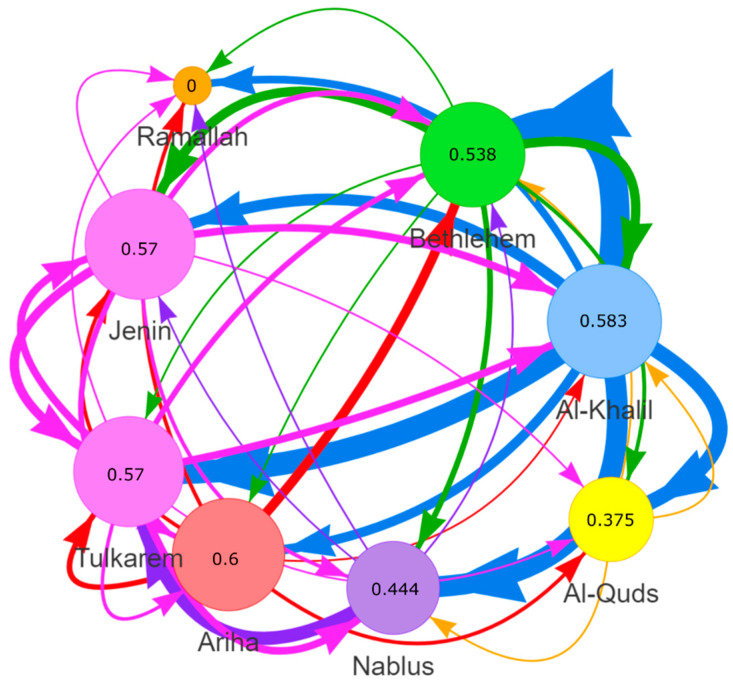
Potential SARS-CoV-2 transmission network based on reported sample location (district). The transmission network was built at StrainHub by mapping the district metadata (Supplementary Table S1) onto a phylogenetic tree in Figure 2. A “source hub ratio, SHR” centrality metric was used. The arrows represent potential transition of SARS-CoV-2, based on the included data, from one location to another location, and line weight reflects the transmission frequency.

The phylogenetic tree of sequenced SARS-CoV-2 genomes (Figure 2), represented as a transmission network (Figure 3), illustrates frequent exchange between the different districts. However, the direction of transmission is unclear from these data. Indeed, transmission links (Figure 3) are based on the available data, and there maybe additional sources or intermediates. This also suggests there is no single identifiable location source of the Delta Variant of SARS-CoV-2 in Palestine. Full contextual data for samples can be found in Supplementary Table S1. The transmission network shows that all Palestinian districts have a SHR value close to 0.5 (0.375–0.6), except the district of Ramallah (SHR = 0), which indicates that all districts are hubs for SARS-CoV-2 except for Ramallah, which is a sink.

**Figure 4:**
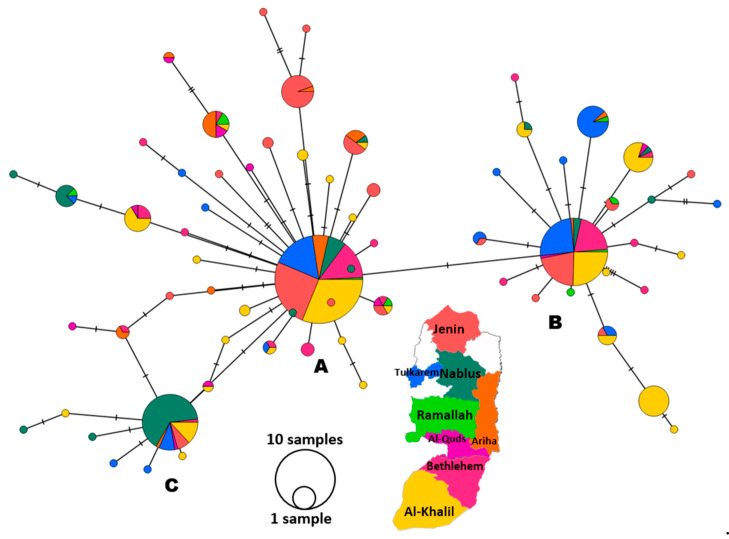
Median-joining haplotype network of the spike region of 462 SARS-CoV2 isolates. Node colors represent district of isolate collection, as indicated in the color map key. Each node represents a haplotype. The diameter of the node circle is proportional to the number of sequences. The number of hatch marks on the connecting lines between nodes indicates the number of mutations between nodes. Three major haplogroups are shown in bold **A**, **B**, and **C**.

However, the haplotype network showed 67 haplotypes forming three major haplogroups, represented by three large circles: A, B, and C (Figure 4). Haplogroup A is the core cluster, containing 38 haplotypes from all locations of the study area. Haplogroup B is the second largest circle in diameter, with 22 haplotypes. Haplogroup C contained only seven haplotypes originating mainly from the Nablus district. Apart from five, all of the differences between adjacent haplotypes are restricted to one SNV, as indicated by the hatch marks. Each haplogroup had a star-like formation around it, representing subhaplogroups (intermediate circles) or even a single haplotype (smallest circles).

### 3.5. Genetic Diversity Analyses

The analyses (Table 3) were based on the three major haplogroups (Figure 4) spun off from the PopArt 1.7 analysis, which utilized 462 sequences of the spike region that were fully sequenced. Meanwhile, the rest (109) were excluded from the total bulk (571), as they were rejected by PopArt 1.7. Cluster A (*n* = 241), the largest of the three, showed the highest number of haplotypes (h) and number of spike mutations (η); however, the ratio of the number of haplotypes to the number of sequences (h:n) remained constant (=0.19) in the three clusters, indicating comparable haplotype distribution across the three clusters. Similarly, the ratio of the number of mutations to the number of sequences for cluster A, B, and C was 0.21, 0.25, and 0.19, respectively. It is obvious that cluster A had the highest Eta:n, which runs parallel with the highest nucleotide diversity (π = 0.0008) and the highest average number of nucleotide differences between any two sequences in the cluster (k = 2.01), specifying cluster A as the most diverse of the three. However, nucleotide diversity (π) (total = 0.0009; ranging = 0.0002–0.0008) is still considered relatively low; simultaneously, haplotype diversity (Hd) (total = 0.87; range = 0.44–0.81) is considered high. High haplotype diversity (Hd) with low nucleotide diversity (π) is indicative of closely related haplotypes. The three neutrality tests, including Tajima’s D, Fu-Li’s F, and Fu-Li’s D, were significantly below zero for individual clusters and the overall bulk (Table 3).

The study showed high levels of genetic differentiation, with Wright’s F-statistic pair genetic differentiation distance (Fst) between any two clusters greater than 0.25 (Fst = 0.47–0.70), together with low (Nm = 0.21) to medium (Nm = 0.36 and 0.57) gene flow between the three SARS-CoV-2 clusters (Table 4). The other genetic differentiation estimators such as the mean coefficient of gene differentiation (Gst) and the number of net nucleotide substitutions per site between populations are relatively low (Gst = 0.16–0.24, Da = 0.000–0.0006) and not in accordance with Fst. In contrast to the elevated Fst statistic, the average number of nucleotide substitutions per site between populations (Dxy), another measure of genetic differentiation, was unexpectedly low (Dxy = 0.0009–0.0011). The average number of nucleotide differences between populations (Kxy) was shown to be low (Kxy = 1.74–2.88). The Hudson–Kreitman–Aguadé (HKA) test was shown to be very low (HKA = 0.000–0.036). The test denotes that under a neutral theory of molecular evolution, it is assumed that polymorphism within a population indicates divergence between populations.

Table 5 shows that the minimum number of recombination events (R_m_) in the three clusters was minimal (R_m_ = 0–1), with negligible recombination events taking place between adjacent sites (R_s_ = 0.007–0.009). However, considerable recombination within the gene (R_g_ = 27–10^5^) was evident.

## 4. Discussion

The Delta Variant, which was first detected in India in October 2020 [36] and spread to more than 170 countries globally (GISAID), was introduced into our region at the beginning of May 2021 [37]. In 2021, Palestine witnessed two COVID-19 peaks: the first was the Alpha Variant peak during March–April, and the second was the Delta Variant during August–October [10,38,39,40]. In mid-September 2021, the number of active COVID-19 cases increased to 32,533, with a mortality rate of 19 per day (3850 deaths), owing to the spread of the Delta Variant. The entire country was enlisted on the list of international high-risk areas [38]. The situation was in line with reports that SARS-CoV-2 genomes from Israeli, Lebanese, and Egyptian patients were dominated by the Delta Variant during July to December 2021 [41,42]. Our study detected 45 cases of the Delta Variant who were Palestinian travelers arriving mainly from Turkey through the border bridge with Jordan and tested for COVID-19 directly upon arrival. However, the purely spatial distribution of COVID-19 cases in Palestine showed eight significant geographical clusters (Figure 1). Phylogenetic analysis (Figure 2) showed the absence of any clear pattern of geographic and genetic clustering (Figure 4). Conversely, haplotype networking displayed three haplogroups, though still without any geographical implications. By contrast, global-level studies have disclosed the SARS-CoV-2 haplotype association with geographic origin and case fatality rates among COVID-19 patients [43]. Compared to a vast geography like India, both analyses showed a high number of haplotypes and lineages, suggesting that the Delta Variant has been imported into and exported from Palestine multiple times [44]. Nearest neighbor analyses suggest that large numbers of Palestinians cross the Palestinian–Israeli borders as close relatives, laborers, or even as regular attendants of the Israeli industrial zones in Palestine and in Turkey, which acts as a regional transport hub. It was clearly observed that the Palestinian and Israeli peaks in 2021 had a sequential pattern in which the Palestinian peaks (maximum number of cases in the highest peak was around 8k) were always preceded by the Israeli ones, with a short period of a couple of weeks to one month in between (maximum number of cases in the highest peak was over 75k) [45] (Supplementary Figure S1). On the other hand, the Jordanian peak in spring was identical to that in Palestine, and the second peak was in December 2021, one month after the second Palestinian peak, ruling out any effect of the Jordanian Delta peak on Palestine, whereas the opposite could have happened [46]. Based on the timing of peaks and the fact that both Palestinians and Israelis are adjacent communities, the transfer of SARS-CoV-2 from the Israelis to the Palestinians is highly probable, including the Delta Variant, especially when knowing that the first Israeli Delta Variant was at the beginning of May, and the first Palestinian Delta Variant was reported two months later, at the end of June 2021 [37,47]. This lag time was enough to effectively spread the Delta Variant in the Palestinian community. Furthermore, the nature of the Delta Variant, which is 63–167% more transmissible and emerges 1.4–2.6 times faster than the Alpha Variant, could have contributed to the spread of the lethal COVID-19 variant [48]. Yet, the recombination between Alpha and Delta SARS-CoV-2 variants is extremely rare; thus, recombination cannot be used to explain the Delta peak that followed the Alpha one [49]. Additionally, in a relatively small geography like the West Bank, the absence of any geographical clustering with a high number of haplotypes and lineages may further indicate active endogenous circulation of the Delta Variant (Figure 3) due to noncompliance with the official preventive regulations such as the lockdown, mass gatherings like weddings, social distancing, and mask wearing. In addition, the transmission network showed that none of the districts formed a source of COVID-19; rather, they were hubs (SHR ≈ 0.5) with equal weights reflected by equal node sizes, except for Ramallah, shown as a sink due to the limited sample size. Although SHR does not indicate which node is the most important in the spread of SAR-CoV-2, Al-Khalil district has the thickest arrows originating from the node (outdegree), indicating a higher frequency of transitions.

In this study, the total nucleotide diversity (π = 0.0009 ± 0.000, Table 3) across the SARS-CoV-2 spike region was relatively high compared to the global nucleotide diversity (π = 0.00044 ± 0.00001) as well as that of the global regions across the whole genome [50]. However, the neutrality tests, Tajima’s D, Fu-Li’s F, and Fu-Li’s F (Table 3), significantly deviated from neutrality to the negative side, leading to a loss of equilibrium. The negativity of neutrality tests can be explained by a recent viral population expansion event or a recent introduction of a new mutation or a group of mutations that have placed themselves in the population and became fixed (selective sweep), or by natural (negative) selection in which deleterious alleles are removed, selectively leading to reduced genetic diversity. However, our results suggest the recent population expansion as the most plausible explanation to the low neutrality test values and low nucleotide diversity (π), along with the excess of rare mutations (negative neutrality tests, Table 3). Both forces, population expansion and excess of rare mutations, resulted in low genetic diversity among the viral populations. Moreover, other minor forces are expected to increase diversity, such as co-circulation and population genetic admixture. These results are in congruence with other studies that showed statistically significantly negative values for neutrality tests [51].

The genetic distance between the three SARS-CoV-2 clusters (Fst > 0.25) and low to intermediate gene flow (Nm) supports the clustering of the genetic diversity, with signs of gene flow between cluster A and B (Nm = 0.57) and between I and C (Nm = 0.36), but with low transfer of genetic diversity (Nm = 0.21) between cluster B and C due to extensive movement of hosts. A study in South America showed statistically significant values, indicating slight genetic differentiation [51]. Unlike the Fst, the other genetic differentiation estimators, Gst and Da, were underestimated due to the high mutation rate [33,52]. Furthermore, the low values of Dxy (0.0009–0.0011) combined with high Fst values can be explained by selection sweep, a mutation that increases its frequency and becomes fixed in a population, which ultimately reduces genetic variation after a period of time. The transmission network (Figure 3) partially supports the potential exchange of genes between clusters due to host movement between districts, especially those coming from Al-Khalil, the most COVID-19 prevalent district, forming the node with the thickest outdegree arrows in the transmission network. More evidently, the value of Rg, the recombination parameter for the entire gene, is high (27–10^5^), suggesting that exchange within the gene may be occurring to varying degrees in the three clusters but more so in cluster C.

The study suggests that the three clusters did not completely evolve into isolated distinct units or clusters, which is most probably due to the recent population expansion, where Fst is expected to increase and Nm to decrease over time. The low estimates of HKA suggest very low departure from neutral theory, indicating an almost constant ratio between within-population polymorphism and between-population divergence, explained by neutral selection of mutations and a low recombination rate [35,53]. In addition to the mutation rate, the recombination rate is a major contributor to genetic diversity in viral genomes. Although a low recombination rate is classically explained by genetic hitchhiking and background selection, the spike gene might not be enough to detect recombination compared to WGS, as previously thought [49,54,55]. Further, the high frequency of consecutive SARS-CoV-2 pandemic waves and the genetically similar viral lineages add up to the difficulty of detecting an accurate recombination rate [49,54]. With the conflicting reports on recombination events in SARS-CoV-2, longer time scales are needed for the recombination to become more pronounced and to take effect on the long-term evolution of the virus [54,56].

As a limitation of the study, sampling did not reflect the incidence scenarios in the Palestinian districts, but rather reflected the preparations and activity in collecting samples as well as the degree of efficiency of the surveillance system in that district. Sampling was underrepresented in districts like Ramallah and absent from districts like Salfit and Qalqilya. This is evident in the transmission network, which identifies Ramallah as a sink of SARS-CoV-2 (Figure 3). Therefore, the potential for sampling bias precludes drawing firm conclusions from such analyses.

## 5. Conclusions

Our study confirmed the utility of genome sequencing analysis in ascertaining and understanding the dynamics of SARS-CoV-2 variants in Palestine in terms of spatial distribution, genetic variation, and forces shaping the creation of new variants, particularly mutation and recombination rates. This study reaffirmed the use of haplotype networking as a complementary technique to phylogenetic analysis, as the latter assumes no recombination, which is not the case in many RNA viruses such as SARS-CoV-2 [57]. This makes haplotype networking a good surrogate, and it is upheld by genetic diversity statistics. In addition, a temporal dimension should be added to accommodate for the rapid increase in the genetic diversity indices over time. The genetic diversity indices of the viral spike region can be amalgamated with vaccine development, in which vaccine efficacy decreases with high genetic diversity, mortality, and morbidity rates, as well as with the clinical picture and virulence. We also stressed the value of sequence deposition in international data repositories in allowing for the evaluation of local epidemiology patterns in a regional and global context.

## Figures and Tables

**Table 1:** Pangolin COVID-19 lineages of the study population, August 2021.

Lineage	Frequency	Percent
B.1.617.2	177	31.00
AY.106	111	19.44
AY.122	90	15.76
AY.121	74	12.96
AY.122.1	32	5.60
AY.112.3	22	3.85
AY.25.1	16	2.80
AY.126	10	1.75
AY.73	6	1.05
AY.124	5	0.88
AY.4.6	3	0.53
AY.46.6	3	0.53
AY.5	3	0.53
AY.4	2	0.35
AY.42	2	0.35
AY.43	2	0.35
AY.45	2	0.35
AY.71	2	0.35
AY.9	2	0.35
AY.111	1	0.18
AY.38	1	0.18
AY.46.4	1	0.18
AY.64	1	0.18
AY.9.2	1	0.18
B.1.1.7	1	0.18
None	1	0.18
TOTAL	571	100.00

**Table 2:** Statistically significant purely spatial COVID-19 clusters.

Locality	District	#Cases/Population	RR	*p*-Value
Beit-Kahil	Al-Khalil	24/4863	8.2	<0.001
Tulkarem city	Tulkarem	57/34,215	2.9	<0.001
Dura	Al-Khalil	33/21,543	2.6	<0.001
Jericho city	Jericho	21/22,293	3.1	<0.001
Jenin city	Jenin	36/26,750	2.2	<0.001
Jalboun	Jenin	6/1508	6.4	0.018
Nour-shams camp	Tulkarem	9/3405	4.2	0.016
Bethlehem city	Bethlehem	22/30,880	2.3	0.022
Ash-Shuhada and Kafr Qoud	Jenin	5/2475, 1/1672	4.7	0.087
At-Taybeh	Jenin	4/2384	5.4	0.254
Zwata	Nablus	4/2715	4.8	0.388
A’nza and Qabatia	Jenin	2/2044, 13/26,396	1.7	0.899
Jalqamous	Jenin	3/2824	3.4	0.959
Beit wazan	Nablus	2/1404	4.6	0.974
Al-Khalil	Al-Khalil	82/221,136	1.2	0.990
Beit Qad	Jenin	2/1655	3.9	0.997

RR, relative risk.

**Table 3:** Genetic diversity indices of the 462 spike sequences of SARS-CoV-2 from Palestine.

	Haplotype–Nucleotide Diversity							Neutrality Tests		
Cluster	N	h	Eta	Hd ± SD	π ± SD	K	S	Tajima’s D	Fu-Li’s F	Fu and Li’s D
Cluster A	241	44	51	0.81 ± 0.08	0.0006 ± 0.000	1.57	50	−2.38 **	−4.55 **	−4.91 **
Cluster B	158	30	40	0.81 ± 0.000	0.0008 ± 0.000	2.01	40	−2.14 *	−5.25 **	−5.98 **
Cluster C	63	13	12	0.44 ± 0.000	0.0002 ± 0.000	0.62	12	−2.15 *	−2.83 *	−2.52 *
Total	462	67	76	0.87 ± 0.01	0.0009 ± 0.000	1.75	74	−2.43 ***	−6.52 **	−8.14 **

N, number of sequences; h, number of haplotypes; Hd, haplotype (gene) diversity; π, nucleotide diversity (per site); K, average number of nucleotide differences between two randomly chosen sequences from within the population; S, number of variable/segregating sites; Eta (η), total number of mutations; ***: *p* < 0.001, **: *p* < 0.01, *: *p* < 0.05 [28,30,31].

**Table 4:** Genetic differentiation indices between the three clusters of the 462 SARS-CoV-2 spike sequences from Palestine.

Pop 1	Pop 2	Fst	Nm	Kxy	Dxy	γst	Da	HKA
Cluster A	Cluster B	0.47	0.57	2.23	0.0011	0.16	0.0005	0.006
Cluster A	Cluster C	0.58	0.36	1.74	0.0009	0.21	0.0005	0.036
Cluster B	Cluster C	0.70	0.21	2.88	0.0014	0.24	0.001	0.000

Fst, Wright’s F-statistics, pairwise genetic distance, Nm, gene flow, and population migration were calculated among populations. Nm = (1 − Fst)/2Fst haploid, Nm = (1 − Fst)/4Fst diploid [32]; Kxy, average number of nucleotide differences between populations; Dxy, the average number of nucleotide substitutions per site between populations; Da, the number of net nucleotide substitutions per site between populations [33]; γst (Gst), coefficient of genetic differentiation [34]; HKA, a test based on neutral theory that a genome exhibits intra-population polymorphism and inter-population divergence [35].

**Table 5:** Recombination events in the three clusters of the 462 SARS-CoV-2 spike sequences from Palestine.

Pop	R_m_	R_g_	R_s_
Cluster A	0.0	>10^5^	--
Cluster B	1.0	29.9	0.009
Cluster C	0.0	27.0	0.007

R, variance of the average number of nucleotide differences between pairs of sequences. Recombination parameter R = 4 Nr, N: population size and r: recombination rate per sequence (per gene); R_s_, recombination parameter between adjacent sites; R_g_, recombination parameter for entire gene; R_m_, minimum number of recombination events in the history of sample [35].

## Data Availability

The data in this study are publicly available in the GISAID database (EPI_ISL_10033368-EPI_ISL_10033935).

## Supplementary Materials

The following supporting information can be downloaded at https://www.mdpi.com/article/10.3390/pathogens13060521/s1: Table S1. Contextual data for the 573 samples included in the study covering the demographic and test result characteristics. Figure S1. Timeline of the COVID-19 pandemic showing Israeli (IL), Palestinian (IL), and Jordanian (JO) peaks. Dates on the figure show the approximate peak date, and the red lines start from the peak on the Israeli timeline and move down through the Palestinian and Jordanian ones.

## Abbreviations

SARS-CoV-2	Severe acute respiratory syndrome coronavirus 2
COVID-19	Coronavirus disease 2019
WGS	Whole-genome sequences
VOC	Variants of Concern
SNV	Single-nucleotide variation
