# Supplementary material for: Epidemiological Characterization and Genetic Variation of the SARS-CoV-2 Delta Variant in Palestine
Source: Pathogens. 2024 Jun 20;13(6):521. doi: 10.3390/pathogens13060521 (PMC11206313; doi:10.3390/pathogens13060521)
Supplement: Supplementary file 1 [file pathogens-13-00521-s001.zip › pathogens-2997600-supplementary.docx]

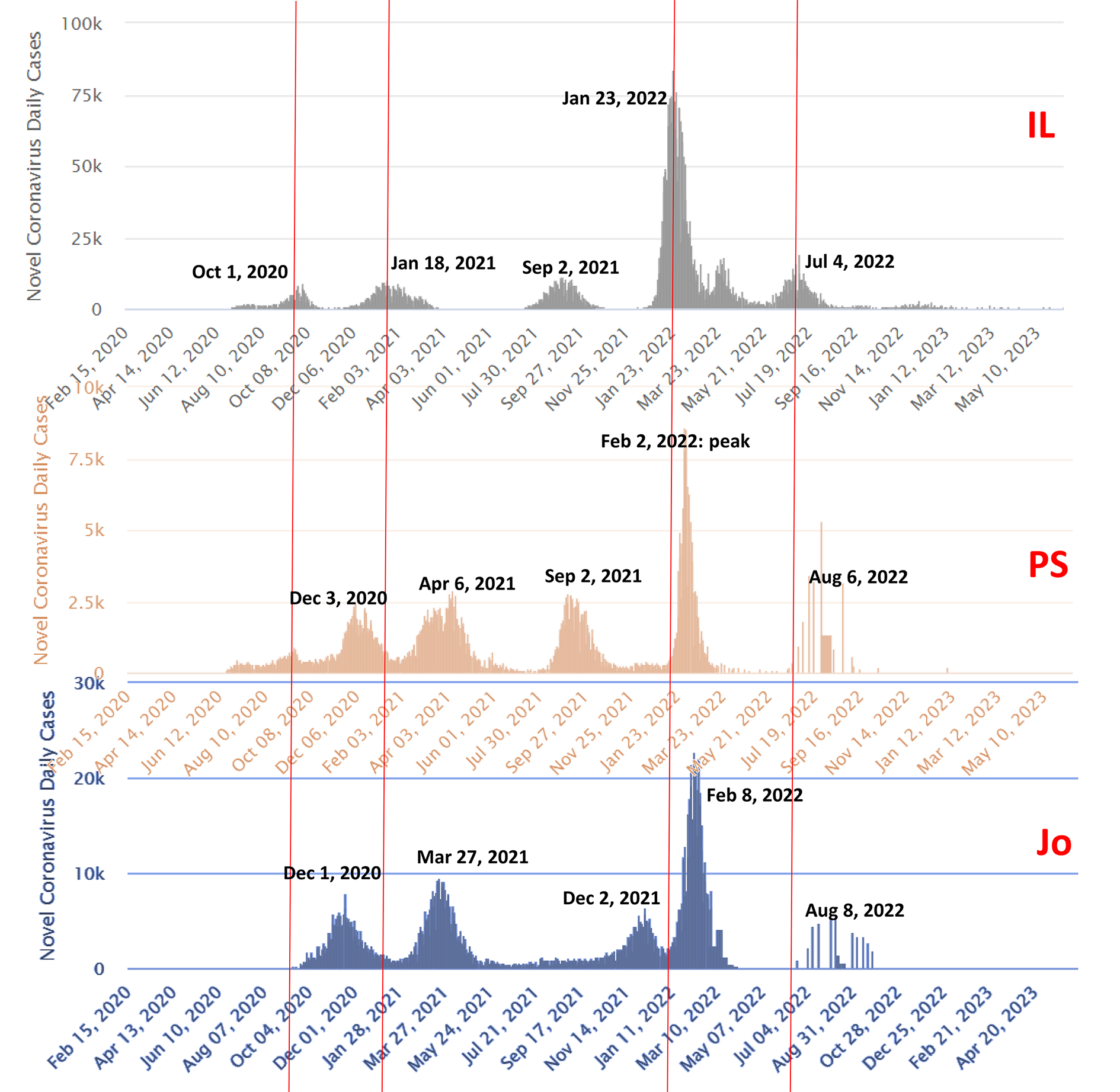


**Figure S1.** Timeline of the COVID-19 pandemic showing Israeli (IL), Palestinian (IL), and Jordanian (JO) peaks. Dates on the figure show the approximate peak date, and the red lines start from the peak on the Israeli timeline and move down through the Palestinian and Jordanian ones.
